# Core Transcription Factors, MicroRNAs, and Small Molecules Drive Transdifferentiation of Human Fibroblasts Towards The Cardiac Cell Lineage

**DOI:** 10.1038/srep40285

**Published:** 2017-01-10

**Authors:** Nicolas Christoforou, Syandan Chakraborty, Robert D. Kirkton, Andrew F. Adler, Russell C. Addis, Kam W. Leong

**Affiliations:** 1 Department of Biomedical Engineering, Duke University, Durham, NC, USA; 2 Department of Biomedical Engineering, Khalifa University, Abu Dhabi, UAE; 3 Department of Biomedical Engineering, Columbia University, New York, NY, USA; 4 Department of Neurosciences, University of California - San Diego, La Jolla, CA, USA; 5 Phelix Therapeutics, Philadelphia, PA, USA

## Abstract

Transdifferentiation has been described as a novel method for converting human fibroblasts into induced cardiomyocyte-like cells. Such an approach can produce differentiated cells to study physiology or pathophysiology, examine drug interactions or toxicities, and engineer cardiac tissues. Here we describe the transdifferentiation of human dermal fibroblasts towards the cardiac cell lineage via the induced expression of transcription factors GATA4, TBX5, MEF2C, MYOCD, NKX2–5, and delivery of microRNAs miR-1 and miR-133a. Cells undergoing transdifferentiation expressed ACTN2 and TNNT2 and partially organized their cytoskeleton in a cross-striated manner. The conversion process was associated with significant upregulation of a cohort of cardiac-specific genes, activation of pathways associated with muscle contraction and physiology, and downregulation of fibroblastic markers. We used a genetically encoded calcium indicator and readily detected active calcium transients although no spontaneous contractions were observed in transdifferentiated cells. Finally, we determined that inhibition of Janus kinase 1, inhibition of Glycogen synthase kinase 3, or addition of NRG1 significantly enhanced the efficiency of transdifferentiation. Overall, we describe a method for achieving transdifferentiation of human dermal fibroblasts into induced cardiomyocyte-like cells via transcription factor overexpression, microRNA delivery, and molecular pathway manipulation.

Transdifferentiation or direct cell reprogramming is the process of converting cells from one specific lineage to a different phenotypically distinct cell type without an intermediate pluripotent stage. This transformation process was first described by Davis *et al*. who used Myod1, a master regulator gene, to convert mouse fibroblasts into skeletal muscle^1^. Since then, transdifferentiation processes have been described for the derivation of neurons, astrocytes, hepatocytes, smooth muscle, and cardiomyocytes^2^,^3^,^4^,^5^,^6^. Unlike Myod1 or Myocd that can solely induce direct cell reprogramming, most other examples of transdifferentiation require the delivery of multiple transcription factors (TF) or microRNAs.

Transdifferentiation *in vitro*, is an alternative to induced pluripotent stem (iPS) cell differentiation, and can produce differentiated cells to study physiology or pathophysiology, examine drug interactions or toxicities, and engineer tissues^7^. Transdifferentiation can also be applied therapeutically *in vivo* by allowing for the *in situ* formation and replacement of cells lost due to disease or injury^8^,^9^,^10^. Importantly, the nascent transdifferentiated cells are autologous and patient-specific eliminating the risk for immuno-rejection^11^. However, for transdifferentiation to be considered as a promising alternative to iPS cell differentiation, it must be both efficient and capable of producing cells that accurately recapitulate native tissue structure and function.

In 2013 three independent groups reported derivation of human induced cardiomyocyte-like (iCML) cells via transdifferentiation. Nam *et al*. used GATA4, Hand2, Tbx5, MYOCD, miR-1, and miR133 to convert neonatal and adult fibroblasts into cardiomyocytes with a small subset of the resulting cells exhibiting spontaneous contractions^12^. Wada *et al*. used GATA4, MEF2C TBX5, Mesp1, and MYOCD to convert human cardiac and dermal fibroblasts into cardiomyocytes that did not spontaneously contract when cultured alone^13^. Finally, Fu *et al*. used GATA4, MEF2C, TBX5, ESSRG, MESP1, MYOCD, and ZFPM2 to transdifferentiate fibroblasts isolated from fetal heart, neonatal skin, or iPS cells into cardiomyocyte-like cells^14^. The overall efficiency of transdifferentiation in all three studies was low (≤25%) which is an important consideration in particular since terminally differentiated cardiomyocytes are non-proliferative. Moreover, the resulting cells did not accurately resemble iPS cell-derived or fetal cardiomyocytes in terms of cytoskeletal organization or capacity to spontaneously contract.

To address the issue of low conversion efficiency, in addition to inducing TF overexpression and delivering microRNAs, various groups have exposed cells undergoing transdifferentiation to small molecules or protein ligands. A Janus kinase 1 inhibitor (JAK1i) significantly increased the conversion efficiency of mouse fibroblasts to cardiomyocytes while using microRNAs^15^. Inhibition of the TGFβ pathway significantly enhanced transdifferentiation when using mouse cells, although, activation of TGFβ was beneficial during conversion of human fibroblasts^14^,^16^,^17^. Exposure to FGF2, FGF10, and VEGF significantly enhanced transdifferentiation of mouse embryonic or tail fibroblasts, as did activation of Akt1/Protein kinase B via exposure to IGF1^18^,^19^.

Here we sought to selectively combine TF overexpression, microRNA delivery, and biochemical signaling pathway manipulation for improving the efficiency of reprogramming human dermal fibroblasts (HDF) into induced iCML cells. First we identified a core set of 5 TF with a cardio-inducing effect and then demonstrated significant enhancement of this effect when co-delivered with two specific microRNAs. We performed gene expression and microarray transcriptome analysis to demonstrate significant upregulation of cardiac genes and pathways associated with cardiac function. A genetically encoded calcium indicator was utilized to study cytoplasmic calcium signaling in iCML cells. Finally, we exposed cells undergoing transdifferentiation to small molecules or protein ligands and examined their capacity to alter the transdifferentiation process.

## Results

### Determining the transdifferentiation-inducing potential of cardiac TF and microRNAs

**Figure 1:**
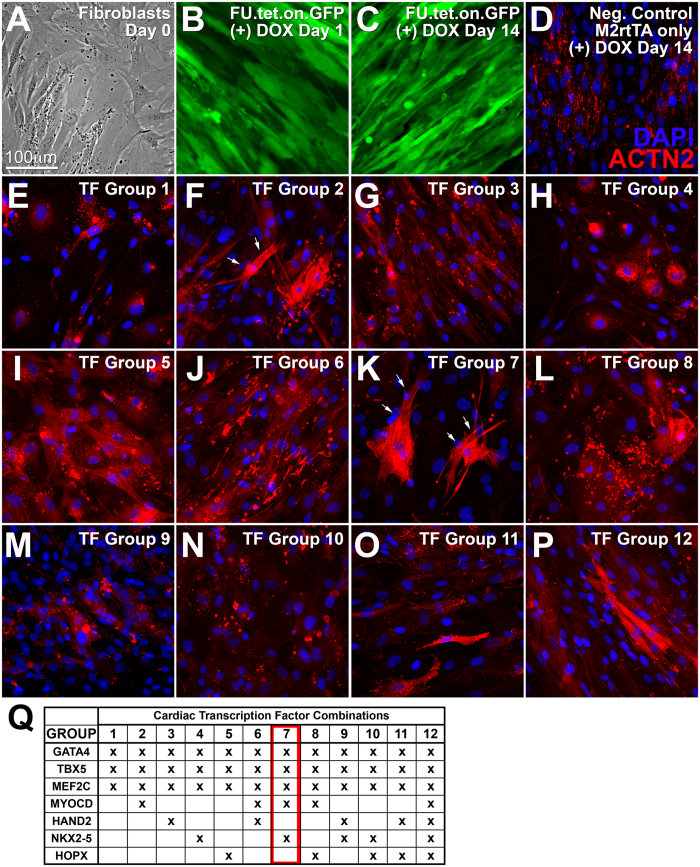
Screen to determine the cardiac transdifferentiation-inducing effect of cardiac TF. (**A**) HDF on day 0 prior to doxycycline addition. (**B,C**) Transduced HDF (M2rtTA and GFP) on day 1 or 14 post doxycycline addition. (**D–P**) HDF transduced with the various combinations of cardiac TF (according to table in panel Q). Expression of cardiac TFs was induced for 10 days. Immunofluorescence imaging for expression of ACTN2. White arrows indicate cells with significant ACTN2 expression in groups 2 and 7. (**Q**) Table detailing combinations of cardiac TFs being overexpressed in HDF.

To determine the effectiveness of our inducible expression system for long-term expression of transgenes, human dermal fibroblasts were transduced with a lentivirus encoding for GFP (Fig. 1A–C). Following induction of expression (via doxycycline addition), GFP^+^ cells were readily detected on culture days 1 and 14. We then tested the capacity of various cardiac TF combinations (Fig. 1Q) to induce transdifferentiation of HDF into ACTN2^+^/TNNT2^+^ cardiomyocyte-like cells. In all combinations tested, three cardiac TF were always included (GATA4, TBX5, MEF2C) as they have been shown to be necessary for the successful transdifferentiation of fibroblasts into cardiomyocytes^4^,^20^. Previously, we showed that culture conditions also play an important role in determining transdifferentiation efficiency. Therefore, in this study we used a low-serum defined culture medium^21^ which was supplemented with a Janus kinase 1 inhibitor (JAK1i) that was shown to significantly enhance transdifferentiation efficiency^15^. We detected ACTN2^+^ cells with substantial cytoskeletal organization throughout their cytoplasm when using cardiac TF combination of GATA4, TBX5, MEF2C, MYOCD (“Group 2”, “GTMM”), GATA4, TBX5, MEF2C, MYOCD, NKX2-5 (“Group 7”, “GTMMN”), GATA4, TBX5, MEF2C, HAND2, HOPX (“Group 11”, “GTMHH”), and GATA4, TBX5, MEF2C, MYOCD, HAND2, NKX2-5, HOPX (“Group 12”, “GTMMHNH”) as shown in Fig. 1F,K,O and P respectively. We detected the highest number of ACTN2+ cells in “Group 7”. When compared to “Group 7”, “Group 2” had 23% ACTN2+ cells, “Group 11” ~12% and “Group 12” ~13%. For all subsequent experiments we used the 5 cardiac TF included in the “Group 7” combination. Significant TNNT2 expression was not detected in all cardiac TF combinations tested. Overall, our preliminary findings suggested that the cardio-inducing effect of TF combinations tested was poor at best, and significant efficiency improvement was necessary to demonstrate definitive derivation of transdifferentiated iCML cells.

**Figure 2:**
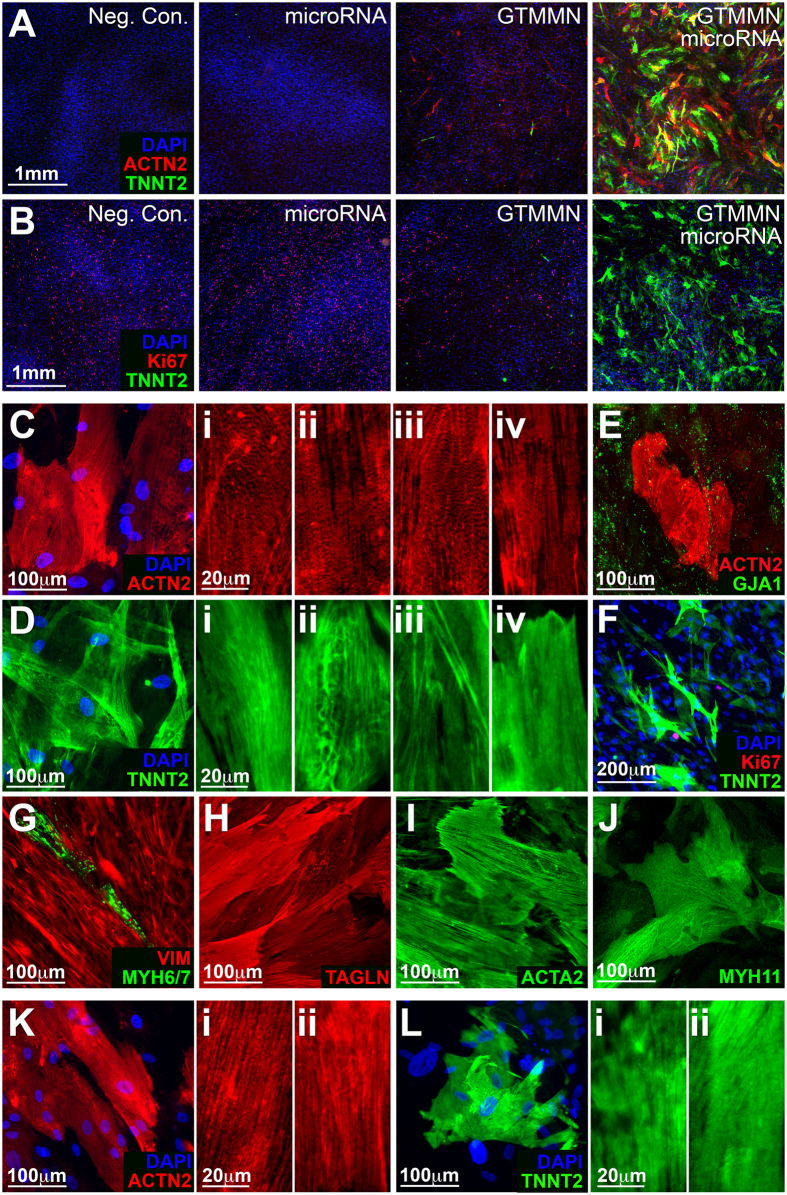
Immunofluorescence characterization of transdifferentiated iCM (cardiac TF and microRNA). (**A**) Expression of ACTN2 and TNNT2 or (**B**) Expression of TNNT2 and Ki67 in HDF transdifferentiated for 2 weeks using induced expression of GATA4, TBX5, MEF2C, MYOCD, NKX2-5 and transfection with hsa-miR-1 and hsa-miR-133a. (Controls: M2rtTA only, microRNA only, cardiac TF only). (**C**) ACTN2. Panels on the right show varying degrees of cytoskeletal organization of ACTN2. (**D**) TNNT2. Panels on the right show varying degrees of cytoskeletal organization of TNNT2. (**E**) ACTN2 and GJA1. (**F**) TNNT2 and Ki67 expression in iCM. (**G**) VIM and MYH6/7. (**H**) TAGLN. (**I**) SMA. (**J**) MYH11. (**K**) ACTN2 expression in iCM 4 weeks following initiation of transdifferentiation. Panels on the right show varying degrees of cytoskeletal organization of ACTN2. (**L**) TNNT2 expression in iCM 4 weeks following initiation of transdifferentiation. Panels on the right show varying degrees of cytoskeletal organization of TNNT2.

Previous studies had demonstrated that microRNAs alone or in combination with cardiac TF induce transdifferentiation of fibroblasts into cardiomyocytes^12^,^15^,^22^. To this end, we tested the capacity of miR-1 and miR-133a to induce HDF transdifferentiation when delivered alone or in combination with “GTMMN”. We determined that a single delivery of miR-1 and miR-133a at the beginning of the process, followed by continuous (2 weeks) induced expression of “GTMMN” significantly enhanced the cardio-inducing effect of the 5 cardiac TF as evidenced by the number of TNNT2^+^ cells detected: 3.8 ± 0.8% versus 0.21 ± 0.04% in “GTMMN” cultures without added microRNAs (p < 0.0001) (Fig. 2A). No TNNT2^+^ cells were detected when using the two microRNAs without “GTMMN” (Supplemental Figure 1). MicroRNA delivery in conjunction with induced cardiac TF expression was associated with a small but significant increase in the number of proliferative cells (Ki67^+^ nuclei): 18.0 ± 5.7% versus 12.0 ± 1.3% in “GTMMN” cultures without added microRNAs (p: 0.031) (Fig. 2B). Based on these findings, in all subsequent experiments we induced cell transdifferentiation using an initial delivery of miR-1 and miR-133a followed by continuous induced expression of “GTMMN”.

The degree of cytoskeletal organization of ACTN2 and TNNT2 in iCML cells 2 weeks post initiation of transdifferentiation was examined using immunofluorescence (Fig. 2C,D). We readily detected a low level of cross-striated ACTN2 in transdifferentiated cells although it varied between cells (Fig. 2Ci–Civ). Similarly, TNNT2 organized in long fibers that transversed the long-axis of the cell (Fig. 2Di–Div) but did not form cross-striations. The cytoskeletal organization of both ACTN2 and TNNT2 was immature and did not accurately resemble the level of organization observed in control cultures of neonatal rat ventricular cardiomyocytes (Supplemental Figure 2). Further culture of transdifferentiated iCML cells (4 weeks) did not result in a substantial change in the degree of cytoskeletal organization of ACTN2 or TNNT2 (Fig. 2K,L). We did not detect ACTN2 or TNNT2 expression in control cell cultures treated only with microRNAs or transduced with M2rtTA alone (Supplemental Figure 1A,G).

Connexin 43 (GJA1) was detected throughout the transdifferentiated cell monolayer surrounding both ACTN2^+^ cells and all other cells (Fig. 2E). This observation was consistent in control cultures (Supplemental Figure 1D,J). Ki67 expression was absent in the nuclei of TNNT2^+^ cells suggesting a lack of proliferation in iCML cells (Fig. 2F). Expression of cardiac myosin heavy chain was rarely detected in iCML cells (Fig. 2G) and was not detected in control cells (Supplementary Figure 1C,I). Expression of smooth muscle markers TAGLN, ACTA2, and MYH11 was detected in cultures of transdifferentiated cells (Fig. 2H–J). While TAGLN and ACTA2 expression was detected in control cells as well, MYH11 was absent (Supplemental Fig. 1E,F,K,L). The expression of smooth muscle genes is likely due to the inclusion of MYOCD in the transdifferentiation cocktail. MYOCD is known to activate both cardiac and smooth muscle transcriptional pathways and has been shown to generate cells that express both cardiomyocyte and smooth muscle markers^5^,^21^. Expression of the three smooth muscle proteins was also detected in cultures of neonatal rat ventricular cardiomyocytes (Supplemental Figure 2F–H).

**Figure 3:**
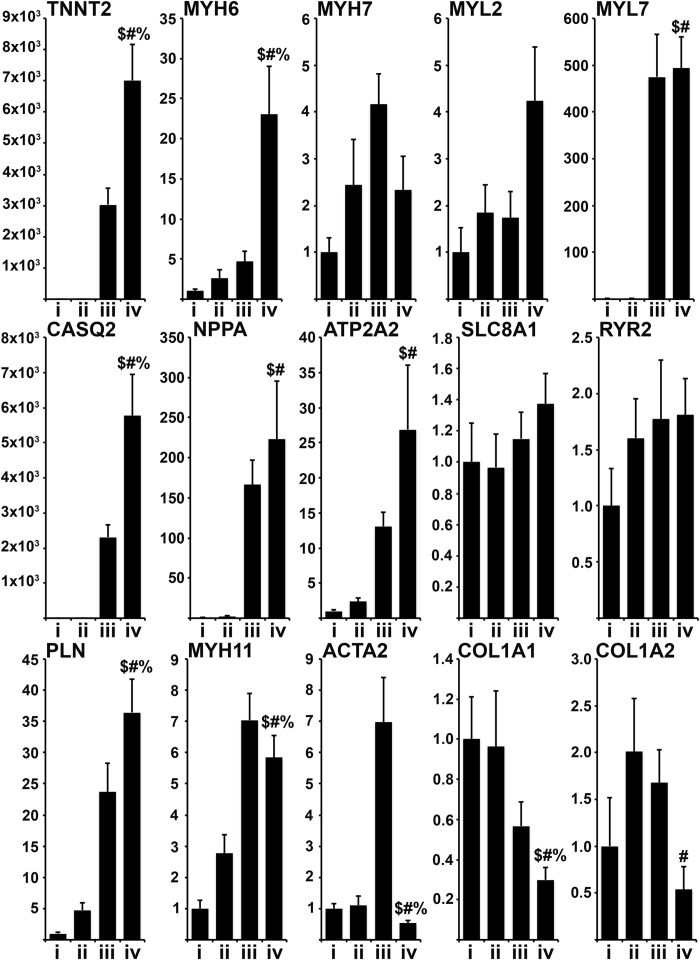
Gene expression analysis. Relative gene expression analysis performed using RT-PCR on RNA isolated from cells being transdifferentiated for 14 days. Samples: (i) Negative Control, (ii) microRNA only, (iii) cardiac TF only, (iv) microRNA and cardiac TF. $ denotes significance compared to group i, # denotes significance compared to group ii,% denotes significance compared to group iii (ΔΔCt analysis, n: 4, Error Bar: Standard Deviation, T-Test: $ or # or % < 0.05).

Gene expression analysis was performed to determine the degree to which the reprogramming process affects transcription as shown in Fig. 3. Transdifferentiation following initial delivery of microRNAs and inducible expression of “GTMMN” for 2 weeks resulted in significant upregulation of TNNT2, MYH6, MYL7, CASQ2, NPPA, ATP2A2, PLN, and MYH11 when compared to negative control cells (M2rtTA vector only). No significant change was detected for MYH7, MYL2, SLC8A1, and RYR2. Fibroblast markers COL1A1 and COL1A2 were downregulated in transdifferentiated cells. When examining the effect of microRNA delivery in cells transdifferentiated under inducible expression of “GTMMN” we detected a significant upregulation in expression levels of TNNT2, MYH6, CASQ2, PLN, and a significant downregulation in expression levels of MYH11 and ACTA2. As expected, expression of “GTMMN” was significantly upregulated in cells where their expression was being induced (Supplemental Figure 3).

### Global Transcriptome and Gene Pathway Analysis

**Figure 4:**
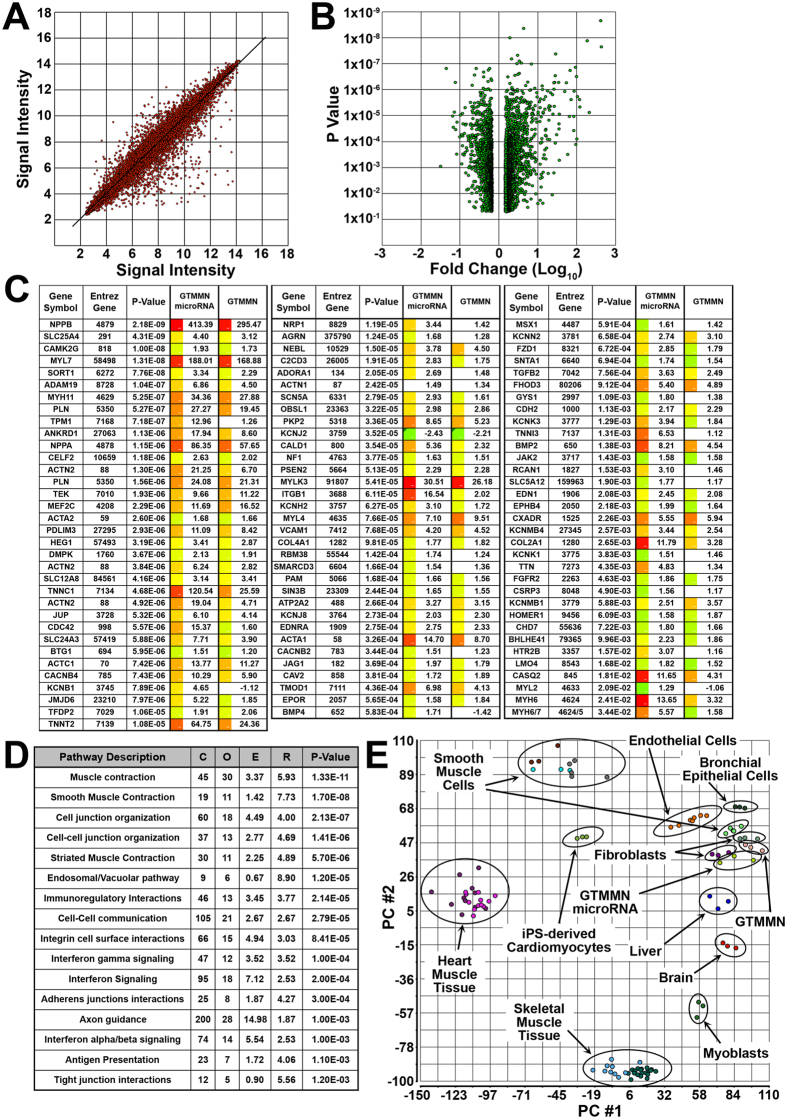
Microarray gene expression analysis. (**A**) Signal intensity plot for individual chip probes when comparing iCM (cardiac TF and microRNA) to control HDF. (**B**) Volcano plot displaying the relationship between P-Value determined using ANOVA versus calculated fold change for each probe. Comparison between iCM (cardiac TF and microRNA) and control HDF. Including only probes with defined fold change (< or >1.5) and significant P-Value (<0.05). (**C**) Fold change and P-Value of selected genes previously described as playing a role in cardiac cell development or function. Fold change is shown for cells transdifferentiated in the presence of cardiac TF and microRNA or cardiac TF only (Red: 413.39, Green: −2.43). (**D**) Molecular pathways associated with significantly upregulated genes when comparing iCM to control HDF as determined by the WEB-based GEne SeT AnaLysis Toolkit (WebGestalt). The “Gene #” column refers to the number of identified genes that belong to a particular pathway and the “P-value” column refers to the P-value of each of the pathways and based on the number of identified genes. (**C**) reference gene number in category, O: number of genes in gene set and category, E: expected number in category, R: enrichment ratio. (**E**) Principal component analysis performed on normalized signal values for each of the chip probes as well as probes from previously published studies.

To further establish the overall effect that microRNA delivery and induced expression of “GTMMN” had on cells undergoing transdifferentiation we performed microarray gene expression analysis. We also analyzed cells being transdifferentiated with induced expression of “GTMMN” alone without microRNA delivery as well as control cells transduced only with M2rtTA and cultured in the same conditions as cells undergoing transdifferentiation. Results indicated that our transdifferentiation process yielded cells exhibiting a variation in the signal intensity for a number of probes (Fig. 4A). Further analysis allowed us to identify probe sets with a change in signal intensity corresponding to a significant increase or decrease in gene expression (p-value < 0.05, fold change<or >1.5) when comparing transdifferentiated cells to control (Fig. 4B and Supplemental Table 1). This enabled us to identify a list of candidate genes that are known to play a role in cardiac development, physiology, and function (Fig. 4C)^23^.

We applied an analytical technique on the entire list of upregulated or downregulated genes, similar to that described by Zhang *et al*., to establish molecular pathways associated with transdifferentiated cells^24^ (Fig. 4D and Supplemental Figure 4). We determined that molecular pathways associated with upregulated genes included: muscle contraction, smooth muscle contraction, cell and cell-to-cell junction organization, and striated muscle contraction. Similar analysis was performed when comparing cells transdifferentiated in the presence or absence of microRNAs and continuous expression of “GTMMN” allowing us to identify significantly upregulated or downregulated genes and pathways enriched in these gene populations (Supplemental Figure 5). We determined that addition of the two microRNAs induced upregulation of genes associated with molecular pathways responsible for striated muscle contraction, cell-to-cell communication, smooth muscle contraction, and cell junction organization.

To further establish the degree to which iCML cells resemble native or mature cardiomyocytes we used principal component analysis and compared the transcriptome of our derived cells to that of cells analyzed in previous studies including cells from skeletal muscle, heart muscle, smooth muscle, endothelium, bronchial epithelium, fibroblasts, liver tissue, brain tissue, myoblasts, and iPS cell-derived cardiomyocytes. We determined that there is a significant difference between the transdifferentiated cells and those isolated from heart muscle tissue or iPS cell-derived cardiomyocytes which can be attributed to the fact that the cell population analyzed was not enriched for iCML cells.

### Characterization of Intracellular Ca^2+^ Signaling

**Figure 5:**
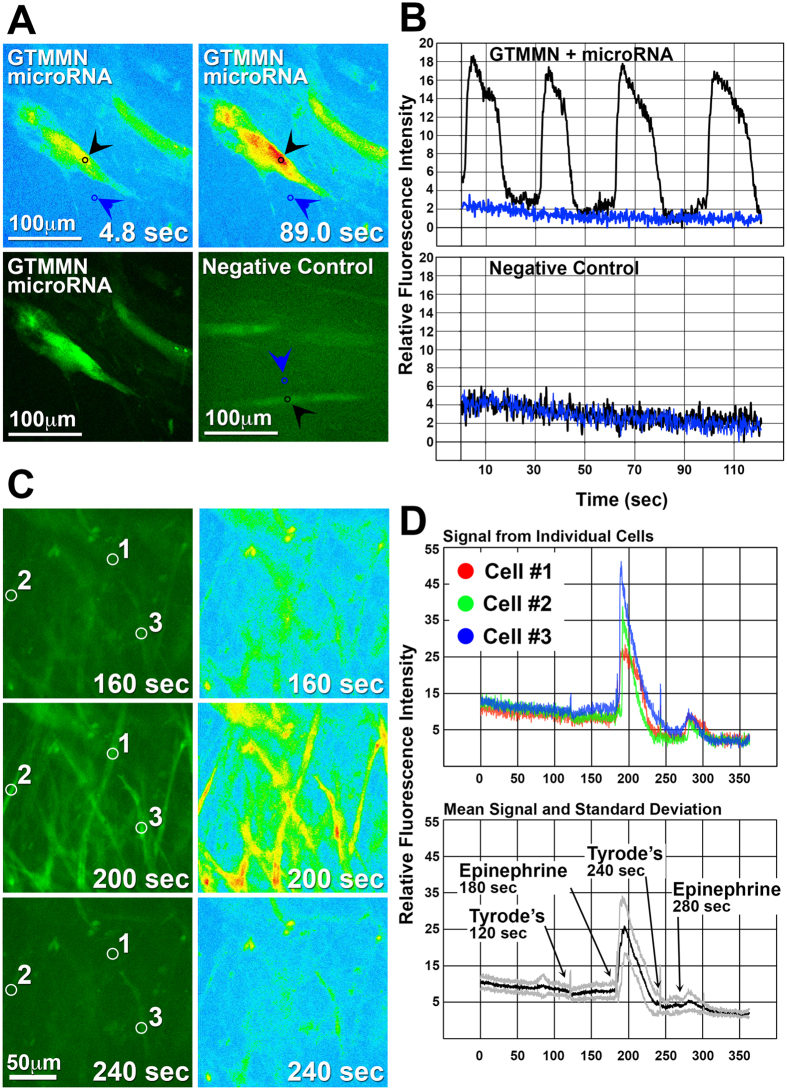
Characterization of intracellular Ca^2+^ activity. iCML cells and control HDF were transduced with a vector allowing the expression of GCaMP3 under the control of the TNNT2 promoter. (**A,B**) Fluorescence intensity imaging of temporal Ca^2+^ transients in an iCML or control cell (M2rtTA only). Black arrows denote the area of a cell at which data acquisition was performed (fluorescence intensity) and blue arrows denote a control area within the same frame. Acquired data was normalized and relative fluorescence intensity is presented in relation with elapsed time. Black and blue line correspond to the arrows in panel A. (**C,D**) Fluorescence intensity imaging of an iCML cell group being stimulated with epinephrine (100 μM). Number coding corresponds to areas within individual cells at which data acquisition was performed (fluorescence intensity). Top graph: Acquired data of individual iCML cells was normalized and relative fluorescence intensity is presented in relation with elapsed time. Bottom graph: The mean value of acquired data from multiple iCML cells (n = 12) is presented as a black line and the standard deviation is presented as the gray lines.

We examined the capacity of iCML cells to generate intracellular Ca^2+^ transients using a genetically encoded calcium indicator (GCaMP3). HDF undergoing transdifferentiation were transduced with a lentivirus allowing the regulated expression of GCaMP3 under the control of the cardiac TNNT2 promoter^21^,^25^. Within 7 days, while observing non-stimulated cells undergoing transdifferentiation we detected rhythmic and regular fluctuations in GCaMP3 fluorescence indicative of spontaneous and long-lasting intracellular Ca^2+^ transients (Fig. 5A,B, Supplemental Video 1). No fluctuations in GCaMP3 fluorescence were detected in the negative control cells. With increasing time in culture (day 12–14) the regular fluctuations in fluorescence intensity observed in non-stimulated cells subsided.

We then used GCaMP3 to test the capacity of iCML cells to respond to adrenergic stimulation by epinephrine application. GFP^+^ cells being monitored using microscopy under physiological conditions (37 °C, 5% CO_2_) were first mechanically stimulated with a drop of Tyrode’s solution followed by epinephrine stimulation (Fig. 5C,D, Supplemental Video 2). This sequence was repeated one more time. Although mechanical stimulation produced a small increase of cytoplasmic Ca^2+^, addition of epinephrine resulted in a very large increase in fluorescence intensity suggesting a relatively quick and large influx of Ca^2+^ in the cytoplasm. This confirmed that the iCML cells express both adrenergic receptors and functional voltage-gated calcium channels, consistent with cardiomyocytes. The effect observed was similar in all cells within the same set of images, although the change in fluorescence amplitude varied. When calculating the mean of fluorescence intensity change, we determined that cells were synchronized in their response (Fig. 5D, lower panel). Despite the large Ca^2+^ influx observed, no contractile activity was detected.

### Determining the effect of small molecule inhibitors and growth factors on transdifferentiation efficiency

**Figure 6:**
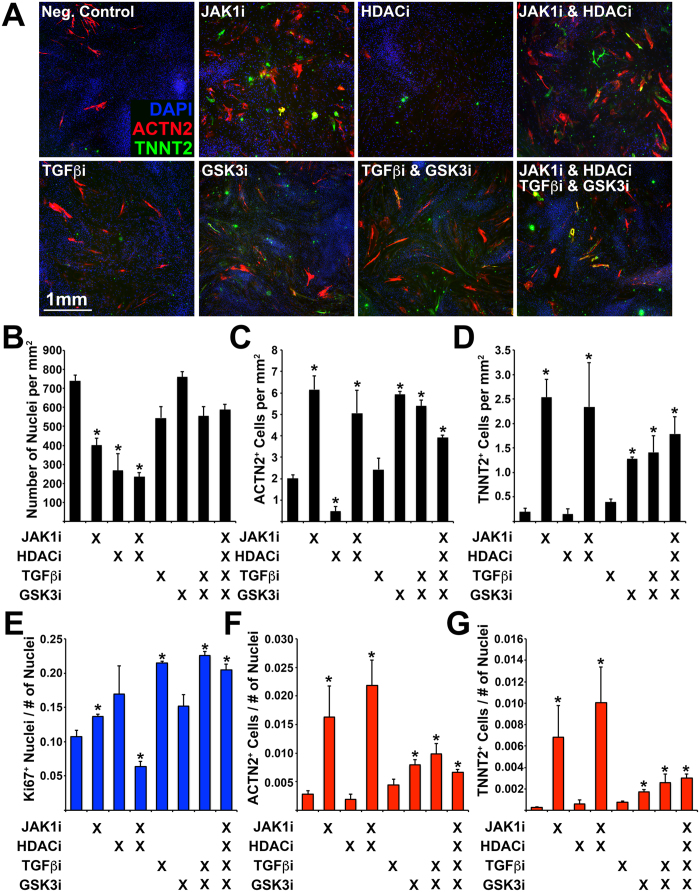
Determining the effect of small molecule inhibitors on transdifferentiation efficiency. (**A**) Immunofluorescence of transdifferentiated cells (ACTN2/TNNT2) during exposure to small molecule inhibitors: Janus protein tyrosine kinase 1 (JAK1i), Sodium Butyrate (HDACi), SB431542 (TGFβi), CHIR99021 (GSK3i). (**B**) Number of nuclei, (**C**) Number of ACTN2^+^ cells, and (**D**) Number of TNNT2^+^ cells (per mm^2^). (**E**) Number of Ki67^+^ nuclei normalized to the total number of nuclei. (**F**) Number of ACTN2^+^ cells normalized to the total number of nuclei. (**G**) Number of TNNT2^+^ cells normalized to the total number of nuclei. Experiment performed in triplicate. 4 images were analyzed for each experiment. Error bar represents calculated standard deviation. Significant difference between two values was calculated using t-test (two-tailed distribution, two sample unequal variance).

Previous studies had demonstrated that inhibition of specific enzymes during transdifferentiation results in significant changes in terms of efficiency^14^,^17^,^18^,^26^. To this end, HDF undergoing transdifferentiation for 2 weeks, were exposed to inhibitors of the following enzymes: Janus protein tyrosine kinase 1 (JAK1i), Histone deacetylase (HDACi), Transforming growth factor-β receptor I and Activin receptor-like kinase 4/5/7 (TGFβi), and Glycogen synthase kinase 3 (GSK3i). We determined their effect on transdifferentiation efficiency by quantifying the total number of nuclei, the number of Ki67^+^ nuclei, and the number of ACTN2^+^ or TNNT2^+^ cells (Fig. 6).

We detected an increase in the absolute number of ACTN2^+^ or TNNT2^+^ cells when using JAK1i alone, JAK1i/HDACi, GSK3i alone, GSK3i/TGFβi, and all four molecules together (Fig. 6A,C,D). HDACi alone resulted in a significant decrease of ACTN2^+^ cells and had no effect in the number of TNNT2^+^ cells. TGFβi alone had no significant effect. When normalizing to the total number of nuclei we determined that the largest increase in transdifferentiation efficiency could be attributed to JAK1i alone, or JAK1i/HDACi (Fig. 6B,F,G). Finally, we observed a significant increase in the number of Ki67^+^ nuclei when using JAK1i alone, TGFβi alone, TGFβi & GSK3i, and all four molecules together. We also observed a significant decrease in the number of Ki67^+^ nuclei when using JAK1i & HDACi (Fig. 6E). We performed the same analysis on cells undergoing transdifferentiation without an initial delivery of microRNAs (Supplemental Figure 6A–F), and determined that significant increase of ACTN2^+^ cells occurred following exposure to HDACi alone, GSK3i alone, and GSK3i/TGFβi, whereas, significant increase in the number of TNNT2^+^ cells occurred following exposure to JAK1i/HDACi (Supplemental Figure 6E,F).

**Figure 7:**
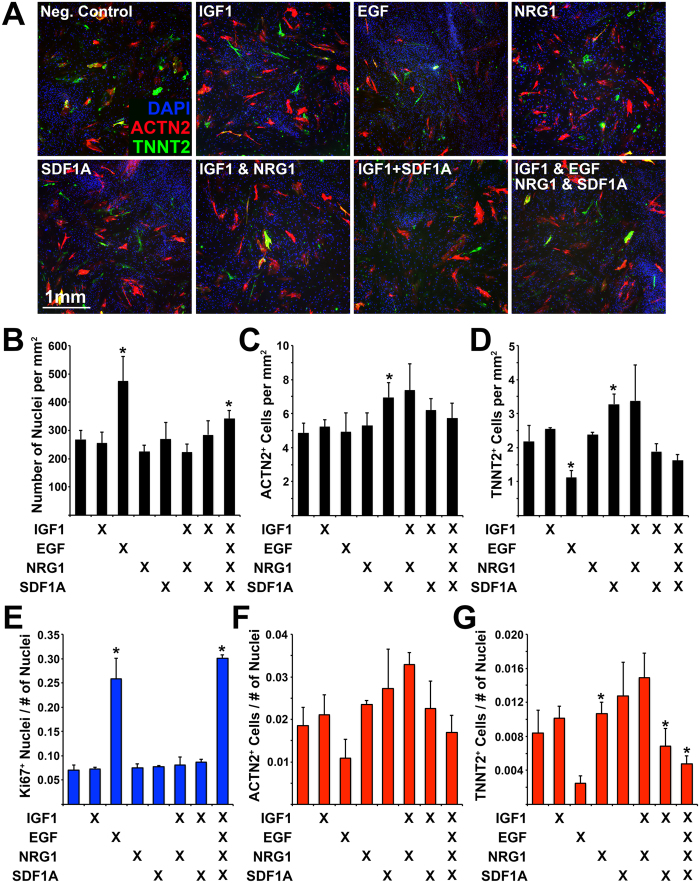
Determining the effect of protein ligands on transdifferentiation efficiency. (**A**) Immunofluorescence of transdifferentiated cells (ACTN2/TNNT2) during exposure to protein ligands: IGF1, EGF, NRG1, SDF1A. (**B**) Number of nuclei, (**C**) Number of ACTN2^+^ cells, and (**D**) Number of TNNT2^+^ cells (per mm^2^). (**E**) Number of Ki67^+^ nuclei normalized to the total number of nuclei. (**F**) Number of ACTN2^+^ cells normalized to the total number of nuclei. (**G**) Number of TNNT2^+^ cells normalized to the total number of nuclei. Experiment performed in triplicate. 4 images were analyzed for each experiment. Error bar represents calculated standard deviation. Significant difference between two values was calculated using t-test (two-tailed distribution, two sample unequal variance).

We also examined the effect that protein ligands previously shown to induce cardiac regeneration or enhance transdifferentiation into the cardiac cell lineage, had upon our cultured cells. This was done in the presence of JAK1i. These ligands included IGF1^18^, EGF, NRG1^27^, and SDF1A^28^ (Fig. 7). We detected a significant increase in the absolute number of ACTN2^+^ or TNNT2^+^ cells when using SDF1A and a significant decrease in the number of TNNT2^+^ cells when using EGF. When normalizing to the total number of nuclei we determined that the only significant increase in the number of TNNT2^+^ cells occurred when using NRG1. As expected, EGF induced a significant increase in the total number of nuclei and in the number of Ki67^+^ nuclei. We also performed the same analysis on cells undergoing transdifferentiation without an initial delivery of microRNAs (Supplemental Figure 6G–L) and determined that SDF1A alone, or IGF1 & SDF1A resulted in a small but significant increase in the number of ACTN2^+^ cells. However, EGF alone, NRG1 alone, IGF1 & NRG1, IGF1 & SDF1A, and all 4 protein ligands together resulted in a significant decrease in the number of TNNT2^+^ cells (Supplemental Figure 6K,L).

## Discussion

Here we describe the transdifferentiation of human dermal fibroblasts into induced cardiomyocyte-like cells via overexpression of 5 cardiac transcription factors and delivery of 2 microRNAs. Having performed an initial screen using various combinations of 7 cardiac TF we identified GATA4, MEF2C, TBX5, MYOCD, and NKX2-5 as capable of inducing significant ACTN2 expression in cells undergoing transdifferentiation, although minimal TNNT2 expression was detected. A single delivery of miR-1 and miR-133a at the beginning of the process was sufficient to induce significant expression of TNNT2. We determined that transdifferentiation efficiency under ideal conditions (low serum, JAK1 inhibitor) resulted in approximately 3.8 ± 0.8% cells being TNNT2^+^ at 2 weeks. Interestingly, addition of the two microRNAs induced a significant decrease in the RNA levels of overexpressed transcription factors GATA4, TBX5, and MEF2C suggesting a stoichiometric correlation between success of the transdifferentiation process (expression of TNNT2) and the concentration of delivered transcription factors. Transdifferentiated cells partially organized ACTN2 in a cross-striated manner whereas minimal organization of TNNT2 was detected. Prolonged culture did not result in an increase in cytoskeletal organization of ACTN2 or TNNT2 and no spontaneous contractions were detected at 4 weeks despite evidence of calcium channel function.

Fu *et al*. used 7 cardiac TF (GATA4, MEF2C, TBX5, ESRRG, MESP1, MYOCD, ZFPM2) to induce transdifferentiation of human iPS cell-derived, dermal, or cardiac fibroblasts into iCML cells^14^. When using dermal fibroblasts as the initial cell source, they determined that 3.71% of cells expressed TNNT2 at 6 weeks. When examining ACTN2 distribution they determined that the cytoskeleton of their iCML cells was partly cross-striated, although no significant organization was detected for TNNT2. They also did not detect any spontaneous contractions in their iCML cells. Wada *et al*. performed a large-scale screen and determined that GATA4, MEF2C, TBX5, MESP1, and MYOCD were the most efficient at converting human cardiac fibroblasts into iCML cells (5.9% TNNT2^+^ cells). When attempting to transdifferentiate human dermal fibroblasts (neonatal foreskin) the authors reported that at 4 weeks they detected ACTN2^+^ and TNNT2^+^ cells however they did not report transdifferentiation efficiency information for these cells. They detected contractile activity in the transdifferentiated cell population only when co-culturing iCML cells with spontaneously contracting mouse cardiomyocytes. Interestingly, we previously reported contractions in cultures of transdifferentiated mouse iCML cells during co-culture with spontaneously contracting rat ventricular myocytes. However we could not determine whether the contractions were due to the movement of neighboring cells or due to the transdifferentiated cells contracting on their own^25^. Finally, Nam *et al*. performed a large-scale screen determining that GATA4, TBX5, HAND2, and MYOCD along with microRNAs miR-1 and miR-133 were the most effective at inducing transdifferentiation of neonatal or adult human foreskin fibroblasts into iCML cells^12^. They argued that miR-1 and miR-133 can directly activate MEF2C expression and thus make its addition dispensable. Under ideal conditions, at 2 weeks they detected 25.1% of the cells being TNNT2^+^. Transdifferentiated cells assembled ACTN2 in cross-striations, although TNNT2 cytoskeletal distribution was not significant. Interestingly the authors initially determined that the yield of TNNT2^+^ cells when using GATA4, TBX5, HAND2, and MYOCD alone was 29% which is higher to that measured when adding microRNAs. No spontaneous contractions were detected in iCML cells derived from dermal fibroblasts.

Each of the 4 studies uses a unique combination of cardiac TF determined to maximize transdifferentiation efficiency of dermal fibroblasts into iCML cells, although a core set of TF is included in all studies: GATA4, MEF2C, TBX5, and MYOCD. All 4 studies describe the derivation of ACTN2^+^/TNNT2^+^ cells, however, no spontaneous contractions were detected in transdifferentiated cells when using human dermal fibroblasts as the initial cell source. Transdifferentiation determinants including upregulation of cardiac genes, downregulation of fibroblast markers, and calcium signaling activation were common in all 4 studies.

Microarray gene expression analysis allowed us to examine the expression pattern of a cohort of cardiac genes in transdifferentiated cells demonstrating significant upregulation when compared to control HDF. Further analysis resulted in identifying several pathways associated with muscle physiology and function. This finding is similar to other studies demonstrating, via microarray gene expression analysis, upregulation of cardiac specific genes^12^,^13^,^14^. We also performed principal component analysis on the entire transcriptome of cells undergoing transdifferentiation and compared it to the transcriptome of a range of tissues or cells. The results clearly showed that there are significant differences between transdifferentiated iCML cells and heart tissue or human iPS cell-derived cardiomyocytes even though we had detected significant upregulation in a large set of cardiac genes. It is important to note that the microarray analysis was performed using RNA isolated from the entire cell population undergoing transdifferentiation. Since only a small subset of cells was ACTN2^+^/TNNT2^+^, it is conceivable that the reason the signal from the entire cell population is similar to that of the starting cell population rather than the target cell population can be attributed to the fact that most of the cells from which RNA was isolated were un-transdifferentiated dermal fibroblasts. A more accurate transcriptome analysis of the transdifferentiated iCML cells would require the prior enrichment and we are planning to do so in our future studies.

JAK1 inhibition resulted in the most significant increase in the number of ACTN2^+^ or TNNT2^+^ iCML cells. This is a similar finding to previous reports describing the pro cardio-inducing effect of JAK1i during the transdifferentiation process of mouse cells into cardiomyocytes^15^,^29^. When inhibiting TGFβ signaling we did not detect a change in transdifferentiation efficiency similar to the finding described by Fu *et al*. who determined that TGFβ inhibition had no effect on the fold change of MYH6^+^ cells, and that addition of TGFβ1 induced a significant increase in transdifferentiation when using only 5 cardiac TF while excluding MYOCD and ZFPM2. In the mouse system, however, TGFβ signaling inhibition was shown to significantly enhance transdifferentiation efficiency suggesting species-specific differences^16^,^17^. We determined that GSK3 inhibition resulted in a significant increase in the absolute number of TNNT2^+^ cells although it also induced significant cell proliferation resulting in a lower normalized number of TNNT2^+^ cells. In a similar study it was determined that GSK3 inhibition induced an increase in transdifferentiation efficiency which, however, was not significant compared to control conditions^14^. We determined that IGF1 exposure had no effect on efficiency, although, two recent studies have shown that Akt/ Protein kinase B and Akt/Phosphoinositol 3-kinase activation promote cardiac reprogramming^18^,^19^. Finally, we determined that exposure to NRG1, a molecule previously shown to induce cardiomyocyte proliferation and repair of the injured myocardium^27^, resulted in a small but significant increase in the number of TNNT2^+^ cells. Exposure of cells to protein ligands was performed in the presence of JAK1i; we did not test the combination of JAK1i + GSK3i + NRG1. When cells, however, were exposed to JAK1i + GSK3i the effect measured was not significantly larger than that measured for JAK1i alone.

We also tested the capacity of an epigenetic regulator inhibitor to affect transdifferentiation. Butyrate, a histone deacetylase inhibitor, has been previously shown to significantly enhance human iPS cell derivation^30^. We determined that butyrate induced a significant decrease in the absolute number of ACTN2^+^ or TNNT2^+^ cells and also resulted in a significant decrease in cell proliferation. Similarly, Ifkovits *et al*. reported that G9a histone methyltransferase inhibition resulted in a decrease in the number of cells activating the TNNT2 promoter element^17^. Moreover, Fu *et al*. reported that inhibition of histone deacetylase or DNA methyltransferase did not induce transdifferentiation^14^. Overall, this suggests that transdifferentiation into iCML cells requires the active form of these enzymes and epigenetic modifications should be performed prior to the induction of transdifferentiation.

Parameters that have been used to determine the overall success of the transdifferentiation process both in our study and the literature have included (1) expression of ACTN2, TNNT2, or TPM1, (2) detection of a cross-striated cytoskeleton, (3) expression or upregulation of cardiac-specific genes, (4) calcium signaling detection and characterization, and (5) electrophysiological characterization^12^,^13^,^14^. Importantly, even though individually these assays suggested that the transdifferentiated cells are cardiomyocytes, no contractile activity was detected when initiating the process using human dermal fibroblasts. A recent report concluded that GATA4, TBX5, and MEF2C were inefficient in achieving a molecular and electrophysiological phenotype resembling mature cardiomyocytes^31^. Overall, it is becoming clear that when using transdifferentiation, significant improvement is necessary to derive functional human cardiomyocytes efficiently.

In conclusion, here we describe an alternative method for transdifferentiating human dermal fibroblasts into induced cardiomyocyte-like cells. We show that a single transfection with miR-1 and miR-133a along with induced expression of GATA4, TBX5, MEF2C, MYOCD, and NKX2-5 activates expression of TNNT2, upregulates a cohort of cardiac-specific genes, and allows generation of intracellular Ca^2+^ transients. Although Ca^2+^ transients were readily detected in iCML cells using a genetically encoded calcium indicator we believe that it is important that future studies utilize more in-depth methods to better characterize the calcium handling properties of the transdifferentiated cells. We also show that JAK1 inhibition, GSK3 inhibition, and NRG1 exposure significantly increase the transdifferentiation efficiency. Although still relatively inefficient at allowing the large-scale production of human cardiomyocytes, this study provides an in-depth analysis of combinatorial approaches for transdifferentiation and further supports the need for developing alternative strategies that significantly improve the efficiency and phenotypic outcomes of current cardiac reprogramming platforms.

## Materials and Methods

### Cell Culture

HDF (Lonza, CC-2509) were plated and expanded in standard growth medium: DMEM – High Glucose, 10% Fetal Bovine Serum (FBS), L-Glutamine, Non-essential amino acids, Sodium Pyruvate, Gentamicin. Expanded cells were aliquoted and frozen at sub-passage 2. For each experiment a fresh aliquot was thawed and used. Cells undergoing transdifferentiation were plated on matrigel-coated substrate as previously described. Transdifferentiation medium was purchased from Lonza (AGM^TM^, CC-3186) and was reconstituted according to the technical specifications without the addition of EGF. Cardiac TF induction of expression was achieved by adding 2 μg/ml Doxycycline (Sigma Aldrich, D9891) to the medium. Under standard conditions, 0.5 μM JAK1 inhibitor (EMD4Biosciences, 420099) and 0.25 mM Sodium Butyrate (303410) was added to the transdifferentiation medium. JAK1 inhibitor and Sodium Butyrate were added in the standard growth medium as per conditions previously described by us^25^. MicroRNAs were purchased from Thermo Scientific: hsa-miR-1 (MIMAT0000416, C-300585-05-0005), and hsa-miR-133a (MIMAT0000427, C-300600-05-0005). MicroRNAs were delivered using reverse transfection according to technical specifications, achieving a final concentration of 50 nM (Lipofectamine RNAiMAX, Life Technologies, 13778-075). Small molecules: SB432542 (10 μM, Tocris, Cat # 1614) CHIR99021 (2 μM, Tocris, Cat # 4423). Protein ligands: All were procured from Prospec Bio and used at a concentration of 10ng/ml. IGF1 (CYT-216), EGF (CYT-217), NRG1 (CYT-407), SDF1A (CHM-262).

### DNA Plasmids, Lentivirus Production, and Cell Transduction

DNA vectors used for lentiviral particle production were prepared as previously described^25^. Briefly, fully sequenced cDNA clones were purchased from Open Biosystems and cloned into an inducible-expression lentiviral vector (Addgene plasmid 19778, gift from Konrad Hochedlinger): GATA4 (BC105108), TBX5 (BC027942), MEF2C (BC026341), MYOCD (BC126307), NKX2-5 (BC025711), HOPX (BC014225), HAND2 (BC101406). To construct the TNNT2.GCaMP3 reporter vector, the ubiquitin promoter-rtTA cassette of FUΔGW-rtTA (Addgene plasmid 19780, gift from Konrad Hochedlinger) was excised and replaced with the human TNNT2 promoter and a Gateway cassette (PCR-amplified from pEF-DEST51, Invitrogen) to create TNNT2-Gateway. The GCaMP3 cassette was cloned into pDONR221 (Invitrogen), and subsequently into TNNT2-Gateway, via Gateway recombination. To construct the FU.tet.on.GFP reporter vector we cloned the GFP fluorescent protein gene in the FU.tet.on inducible expression lentiviral vector. The FUW.M2rtTA lentiviral vector was acquired from Addgene (Addgene plasmid 20342, gift from Rudolf Jaenisch).

Lentiviral particle production was performed as previously described^25^. Briefly HEK293T cells were maintained and expanded using standard growth medium in T-75 tissue culture flasks. Cells were allowed to reach 90% confluence at which point they were transfected in the presence of Opti-MEM^®^ (Life Technologies) with a total of 24 μg of the three lentiviral vectors (12 μg expression vector, 7.7 μg of psPAX2 (Addgene plasmid 12660) and 4.3 μg of pMD2.G(Addgene plasmid 12559)) using Lipofectamine 2000 (Life Technologies). The supernatant containing viral particles was collected at 24, 48, and 72 hours following initial transfection. The supernatant was aliquoted, stored at 4 °C and used within 1 week following collection.

Cells were plated at a concentration of 10^5^ cells/cm^2^ and the next day transduced using supernatant containing viral particles. 6-well plates: 0.5 ml of supernatant for each cardiac TF and M2rtTA. 24-well plates: 0.125 ml of supernatant for each cardiac TF and M2rtTA. The next day the medium was exchanged with fresh medium. Two days following viral transduction the cells were either induced to transdifferentiate *in situ* or enzymatically dissociated and plated into new plates. The transduction medium was supplemented with 8 μg/ml Sequabrene (Sigma Aldrich, S2667).

### Gene Expression Analysis/Quantitative RT-PCR

Primer design was performed using NCBI primer-BLAST. To avoid amplification of non-specific DNA, when applicable, primers were required to span an exon-exon junction and the primer pair was to be separated by at least one intron on the corresponding genomic DNA (Supplemental Table 2). Total RNA was isolated using the RNeasy Mini kit (Qiagen, 74104). Quantitative RT.PCR analysis was performed on a 7900HT real time thermocycler using the QuantiTect SYBR Green one-step RT.PCR kit (Qiagen, 204243). The SDS software (ABI, version 2.4) was used to analyze the data and additional analysis was performed on Microsoft Excel. Relative quantification was performed using the ΔΔCt method and statistical significance was determined using the T-Test. The final reaction product was run on an agarose gel to determine whether the size of the amplimer detected and quantified was the expected.

### Microarray Gene Expression Analysis

HDF were induced to transdifferentiate for 2 weeks. Group 1: Cardiac TF and microRNA, Group 2: Cardiac TF only, Group 3: Control (M2rtTA only). Conversion occurred using transdifferentiation medium supplemented with JAK1 inhibitor and sodium butyrate. Total RNA was isolated using the RNeasy Mini kit (Qiagen, 74104). Its quality was assessed using the Agilent 2100 Bioanalyzer G2939A (Agilent Technologies, Santa Clara, CA)) and Nanodrop 8000 spectrophotometer (Thermo Scientific/Nanodrop, Wilmington, DE). Hybridization targets were prepared with MessageAmp™ Premier RNA Amplification Kit (Applied Biosystems/Ambion, Austin, TX) from total RNA, hybridized to GeneChip^®^ Human Genome U133A 2.0 arrays in Affymetrix GeneChip^®^ hybridization oven 645, washed in Affymetrix GeneChip^®^ Fluidics Station 450 and scanned with Affymetrix GeneChip^®^ Scanner 7 G according to standard Affymetrix GeneChip^®^ Hybridization, Wash, and Stain protocols. (Affymetrix, Santa Clara, CA). This work was performed at the Duke University microarray core facility. The data was submitted to the NCBI GEO repository with accession number GSE81723.

Data analysis was performed as previously described^5^. Briefly we imported the CEL files into the Partek Genomics Suite and normalized it using the RMA algorithm. We performed an ANOVA statistical analysis on the entire data set searching for significant differences between transdifferentiated iCM (5 cardiac TF and microRNA) and control HDF. Significantly upregulated or downregulated genes were identified based on the fact that p-value < 0.05 and Fold Change< or >1.5. Identification of molecular pathways associated with significantly upregulated or downregulated genes was performed using the WEB-based GEne SeT AnaLysis Toolkit (WebGestalt)^24^. Principal component analysis was performed with additional control data files from previously published studies that were uploaded on NCBI Gene Expression Omnibus or EMBL-EBI Array Express: Heart muscle tissue (GSE1145, GSE29819), iPS-derived cardiomyocytes (GSE60293), Smooth muscle cells (GSE11917, GSE11367, GSE29881, GSE29955), Endothelial cells (GSE10804), Bronchial cells (GSE62769), Fibroblasts (GSE34309), Skeletal muscle tissue (GSE36297, GSE31243), Liver (E-TABM-1091), Brain (E-TABM-1091), Myoblasts (E-TABM-1091).

### Ca^2+^ Signaling

HDF were plated in 35 mm plates at a concentration of 10^5^ cells/cm^2^. The next day cells were transduced with viral particles allowing inducible expression of GATA4, TBX5, MEF2C, MYOCD, NKX2-5. Two days later cells were transfected with microRNAs using Lipofectamine RNAiMAX (50 nM final concentration). The next day induction of cardiac TF expression was initiated using doxycycline in transdifferentiation medium containing JAK1 inhibitor and sodium butyrate. Two days later cells were further transduced with a lentivirus allowing expression of GCaMP3 under control of the TNNT2 promoter element.

For Ca^2+^ transient detection, plates were mounted onto a Nikon Eclipse TE2000 Inverted Fluorescent Microscope and maintained in an environmental chamber set to 37 °C. Cells were incubated in Tyrode’s medium (135 mM NaCl, 5.4 mM KCl, 5 mM HEPES, 5 mM D-Glucose, 0.33 mM NaH_2_PO_4_, 1.8 mM CaCl_2_ and 1 mM MgCl_2_; pH 7.4) during all imaging and stimulation steps. Cells in each plate were stimulated by direct addition of a drop of Tyrode’s solution (mechanical control), or 100 μM epinephrine. For each plate, the intensity of GCaMP3 fluorescence signal was recorded following exposure to each stimulus and it was subsequently compiled into a single video file using the Nikon NIS Elements software.

To determine the relative change in calcium concentration within stimulated cells we analyzed the recorded video files using ImageJ. The area of selected responsive cells and the background was individually highlighted within each video file and the fluorescence intensity of the highlighted areas at each frame was measured throughout the time course of the video. Fluorescence intensity was normalized and relative fluorescence intensity was graphed in relation to time. RGB images were created using ImageJ to highlight peak fluorescence in concordance with fluorescent intensity graphs.

### Immunofluorescence and Image Analysis

Prior to immunofluorescence staining, cells were fixed using 2% paraformaldehyde (EMS, RT-15713), permeabilized using 0.2% Triton X-100 (Sigma Aldrich, 93443), and blocked using 10% FBS. Primary antibodies were added for 30 min, followed by three washes, and secondary antibodies for an additional 30 min. All steps were performed at room temperature. Primary antibodies used: anti-ACTN2 (Sigma Aldrich, A7811), anti-TNNT2 (R&D Systems, MAB1874), anti-Ki67 (eBiosciences, 14-5699-82, Abcam, ab15580), anti-GJA1 (Santa Cruz, SC-9059), anti-VIM (Sigma Aldrich, C9080), anti-TAGLN (Abcam, ab14106), anti-MYH6/7 (Abcam, ab15, DHSB, MF20), anti-ACTA2 (Abcam, ab7817), anti-MYH11 (Abcam, ab683). Secondary antibodies used: Raised in chicken or goat against mouse or rabbit antibodies (LifeTechnologies). Conjugated to Alexa Fluor^®^ 568 or Alexa Fluor^®^ 488 (green). Cell nuclei were detected using DAPI (Life Technologies, D1306). Fluorescent cell imaging was performed on a Nikon Eclipse TE2000-U using a Roper Scientific CoolSnap HQ camera and the NIS Elements software suite.

Image analysis and quantification was performed using ImageJ (https://imagej.nih.gov/ij/). Imported images were first converted to 8-bit binary files using the “Threshold” option. We also applied the “Watershed” binary tool to ensure that overlapped events were counted separately. Individual events (DAPI^+^ nuclei or Ki67^+^ nuclei) were quantified using the analyze image function of ImageJ. Individual ACTN2^+^ or TNNT2^+^ cells were manually counted. Operator was not blinded to the experiments.

## Additional Information

**How to cite this article**: Christoforou, N. *et al*. Core Transcription Factors, MicroRNAs, and Small Molecules Drive Transdifferentiation of Human Fibroblasts Towards The Cardiac Cell Lineage. *Sci. Rep.* **7**, 40285; doi: 10.1038/srep40285 (2017).

**Publisher's note:** Springer Nature remains neutral with regard to jurisdictional claims in published maps and institutional affiliations.

## Supplementary Material

Supplemental Video 1

Supplemental Video 2

Supplementary Information

Supplemental Table 1
