# Supplementary material for: Core Transcription Factors, MicroRNAs, and Small Molecules Drive Transdifferentiation of Human Fibroblasts Towards The Cardiac Cell Lineage
Source: Sci Rep. 2017 Jan 10;7:40285. doi: 10.1038/srep40285 (PMC5223186; doi:10.1038/srep40285)
Supplement: Supplementary Information [file srep40285-s3.pdf]

Supplementary Information

TITLE

Core Transcription Factors, MicroRNAs, And Small Molecules Drive Transdifferentiation Of Human Fibroblasts Towards The Cardiac Cell Lineage

AUTHORS & AFFILIATIONS

Nicolas Christoforou[1,2,#], Syandan Chakraborty[3,#], Robert D. Kirkton[1], Andrew F. Adler[4], Russell C. Addis[5], Kam W. Leong[3].

[1]Department of Biomedical Engineering, Duke University, Durham, NC, USA

[2]Department of Biomedical Engineering, Khalifa University, Abu Dhabi, UAE

[3]Department of Biomedical Engineering, Columbia University, New York, NY, USA

[4]Department of Neurosciences, University of California - San Diego, La Jolla, CA, USA

[5]Phelix Therapeutics, Philadelphia, PA, USA

[#]These authors have equally contributed to this work

CORRESPONDING AUTHORS

Kam W. Leong, Department of Biomedical Engineering, Columbia University, Mail Code 8904, 1210 Amsterdam Avenue, New York, NY, 10027, USA, Phone: +1.212.854.8725, Email: kwl2121@columbia.edu

Nicolas Christoforou, Department of Biomedical Engineering, Khalifa University, P. O. Box 127788, Abu Dhabi, UAE, Phone: +973.2.501.8560, Email: nicolas.christoforou@kustar.ac.ae, and Department of Biomedical Engineering, Duke University, 136 Hudson Hall, Box 90281, Durham, NC, 27708, USA, Email: nc28@duke.edu

Contents:

1) **Supplementary Figure Legends**
2) **Supplementary Table Legends**
3) **Supplementary Movie Legends**
4) **Supplementary Figures**
5) **Supplementary Table 2**

Supplementary Figure Legends:

Supplementary Figure 1. Immunofluorescence of control cells. Cells were cultured in the same conditions and for the same amount of time used to derive iCM. (A-F) Negative Control (M2rtTA only), (G-L) Cells transfected only with microRNAs.

Supplementary Figure 2. Immunofluorescence of rat neonatal cardiomyocytes. Cells were cultured in the same conditions and for the same amount of time used to derive iCM. Immunofluorescence for: (A) ACTN2, (B) TNNT2, (C) TNNT2 and GJA1, (D) ACTN2 and SERCA2, (E) RYR2 and TNNT2, (F) TAGLN, (G) ACTA2, (H) MYH11.

Supplementary Figure 3. Gene expression analysis. Relative gene expression analysis performed using RT-PCR on RNA isolated from cells being transdifferentiated for 14 days. Samples: (i) Negative Control, (ii) microRNA only, (iii) cardiac TF only, (iv) microRNA and cardiac TF. $ denotes significance compared to group i, # denotes significance compared to group ii, % denotes significance compared to group iii (T-Test: $ or # or % < 0.05).

Supplementary Figure 4. Microarray gene expression analysis. Pathway analysis for genes that were determined to be significantly upregulated or downregulated when comparing iCM (cardiac TF and microRNA) to negative control HDF. Determined using the WEB-based GEne SeT AnaLysis Toolkit (WebGestalt). The “Gene #” column refers to the number of identified genes that belong to a particular pathway and the “P-value” column refers to the P-value of each of the pathways and based on the number of identified genes. C: reference gene number in category, O: number of genes in gene set and category, E: expected number in category, R: enrichment ratio.

Supplementary Figure 5. Microarray gene expression analysis. Comparison of cells transduced with cardiac TF / microRNA and cells transduced only with cardiac TF. (A) Significantly upregulated, and (B) Significantly downregulated genes in iCM (cardiac TF and microRNA) and

compared to cells transduced only with cardiac TF. (C) Pathway analysis for genes that were determined to be significantly upregulated or downregulated when comparing iCM (cardiac TF and microRNA) to cells transduced only with cardiac TF. Determined using the WEB-based GEne SeT AnaLysis Toolkit (WebGestalt). The “Gene #” column refers to the number of identified genes that belong to a particular pathway and the “P-value” column refers to the P-value of each of the pathways and based on the number of identified genes. C: reference gene number in category, O: number of genes in gene set and category, E: expected number in category, R: enrichment ratio.

Supplementary Figure 6. Effect of small molecule inhibitors or protein ligands on the transdifferentiation efficiency of HDF transduced with 5 cardiac TF only. (A-F) Small molecule inhibitor effect and (G-L) protein ligand effect. (A or G) Number of nuclei, (B or H) Number of ACTN2$^+$ cells, and (C or I) Number of TNNT2$^+$ cells (per mm^2). (D or J) Number of Ki67$^+$ nuclei normalized to the total number of nuclei. (E or K) Number of ACTN2$^+$ cells normalized to the total number of nuclei. (F or L) Number of TNNT2$^+$ cells normalized to the total number of nuclei. Experiment performed in triplicate. Error bar represents calculated standard deviation. Significant difference between two values was calculated using t-test (two-tailed distribution, two sample unequal variance).

Supplementary Table Legends:

Supplementary Table 1. List of genes that are significantly upregulated or downregulated when comparing transdifferentiated iCM (5 cardiac TF and microRNA) to negative control HDF. Also included is the fold change for the comparison between cells transduced with 5 cardiac TFs and the negative control HDF.

Supplementary Table 2. Primers used for gene expression analysis.

Supplementary Movie Legends:

Supplementary Movie 1. Transdifferentiated iCM transduced with a vector allowing the expression of a genetically encoded calcium indicator (GCaMP3) under the control of the TNNT2 promoter element. Cells are not being stimulated and are just passively being monitored using fluorescence microscopy. The video is presented at 10x acceleration.

Supplementary Movie 2. Transdifferentiated iCM transduced with a vector allowing the expression of a genetically encoded calcium indicator (GCaMP3) under the control of the TNNT2 promoter element. Cells are being stimulated with epinephrine at specific time-points (120 sec: Tyrode's, 180 sec: Epinephrine, 240 sec: Tyrode's, 280 sec: Epinephrine). The video is presented at 10x acceleration.

Supplementary Figures:

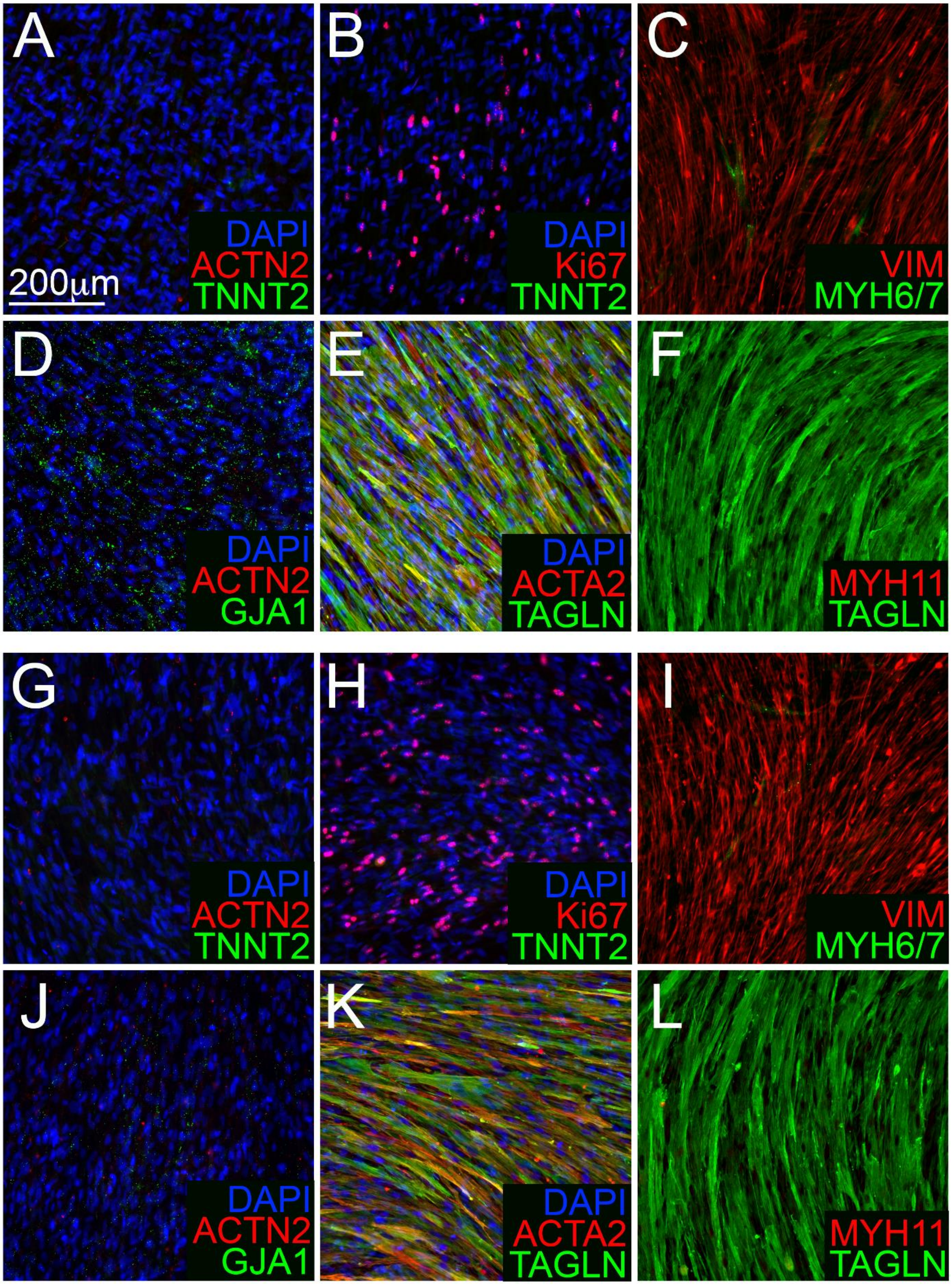

Supplementary Figure 1. Immunofluorescence of control cells.

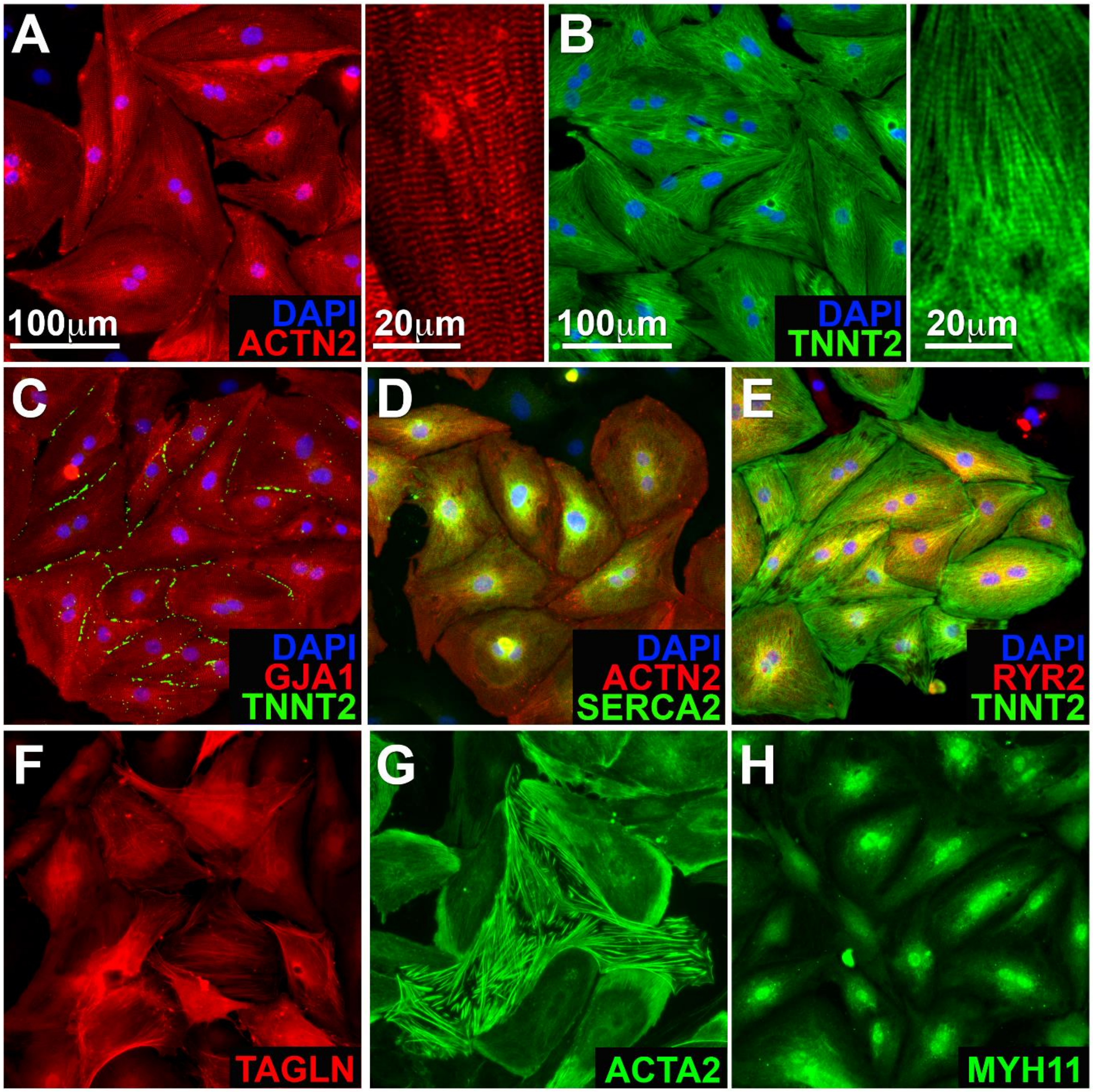

Supplementary Figure 2. Immunofluorescence of rat neonatal cardiomyocytes.

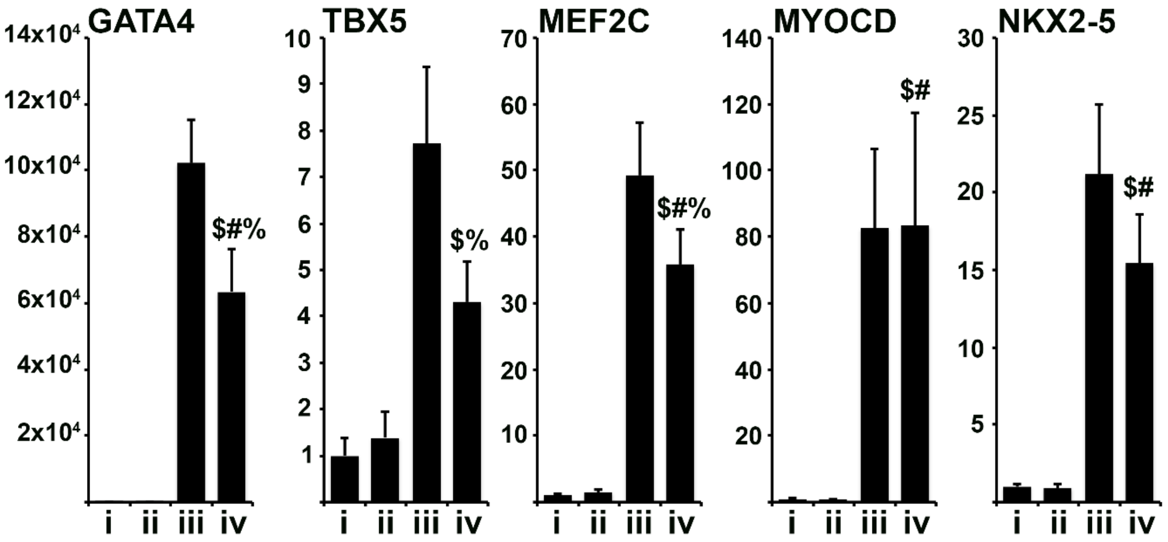

Supplementary Figure 3. Gene expression analysis.

Pathway Commons Analysis: GTMMN+microRNA Versus Negative Control (Upregulated Genes)						
**Pathway Description**	**Entrez Gene**	**C**	**O**	**E**	**R**	**P-Value**
**Muscle contraction**	7134 10398 7137 10580 7169 1674 25802 88 72 4629 7111 4635 800 4638 462159 7139 4624 7168 7414	**45**	**30**	**3.37**	**5.93**	**1.33E-11**
**Smooth Muscle Contraction**	10398 10580 7169 25802 72 4629 800 4638 59 7168 7414	**19**	**11**	**1.42**	**7.73**	**1.70E-08**
**Cell junction organization**	1002 2318 3728 3655 50848 23705 1006 84612 3909 999 9 459 1012 5584 36881001 1000 51208 56288	**60**	**18**	**4.49**	**4.00**	**2.13E-07**
**Cell-cell junction organization**	1002 3728 50848 23705 1006 84612 999 1012 5584 1001 5 1208 1000 56288	**37**	**13**	**2.77**	**4.69**	**1.41E-06**
**Striated Muscle Contraction**	7134 7137 7169 1674 88 7111 4635 4621 7139 4624 7168	**30**	**11**	**2.25**	**4.89**	**5.70E-06**
**Endosomal/Vacuolar pathway**	3133 3134 3105 3106 3135 3107	**9**	**6**	**0.67**	**8.90**	**1.20E-05**
**Immunoregulatory interactions between Lymphoid & non-Lymphoid cells**	718 3133 3134 10666 3105 1525 3689 3106 999 3688 3135 7412 3107	**46**	**13**	**3.45**	**3.77**	**2.14E-05**
**Cell-Cell communication**	1002 2318 3728 3655 50848 23705 88 1006 84612 3909 99 9 9459 1012 5584 36881001 4684 1000 51208 56288 6709	**105**	**21**	**2.67**	**2.67**	**2.79E-05**
**Integrin cell surface interactions**	8516 3655 50848 3689 5770 999 3679 3912 1280 3690 367 3 3688 7412 3680 3911	**66**	**15**	**4.94**	**3.03**	**8.41E-05**
**Interferon gamma signaling**	3133 3134 3105 3106 5770 2634 3135 2210 7412 3717 310 7 4684	**47**	**12**	**3.52**	**3.52**	**1.00E-04**
**Interferon Signaling**	4599 3133 3134 3437 3105 9636 3433 3106 5770 3690 263 4 3135 2210 3717 74123107 3434 4684	**95**	**18**	**7.12**	**2.53**	**2.00E-04**
**Adherens junctions interactions**	1002 3728 23705 1006 999 1012 1001 1000	**25**	**8**	**1.87**	**4.27**	**3.00E-04**
**Axon guidance**	10398 7223 375790 4651 10486 5604 7430 9723 10570 21 4 785 9037 4629 272895788 3983 286 100133941 998 783 3912 3690 3688 6478 23122 8829 4684 6709	**200**	**28**	**14.98**	**1.87**	**1.00E-03**
**Interferon alpha/beta signaling**	4599 3133 3134 3437 3105 9636 3433 3106 5770 3690 263 4 3135 3434 3107	**74**	**14**	**5.54**	**2.53**	**1.00E-03**
**Antigen Presentation: Folding, assembly & peptide loading of class I MHC**	3133 3134 6890 3105 3106 3135 3107	**23**	**7**	**1.72**	**4.06**	**1.10E-03**
**Tight junction interactions**	50848 84612 5584 51208 56288	**12**	**5**	**0.90**	**5.56**	**1.20E-03**

Pathway Commons Analysis: GTMMN+microRNA Versus Negative Control Downregulated Genes)						
**Pathway Description**	**Entrez Gene**	**C**	**O**	**E**	**R**	**P-Value**
**Activation of the pre-replicative complex**	8318 4173 4175 55388 5558 5427 4174 990 1017 6119 5557	**29**	**11**	**2.26**	**4.88**	**5.57E-06**
**Biosynthesis of the N-glycan precursor & transfer to a nascent protein**	8818 79796 9945 29880 29929 6675 79868 5238 29926	**21**	**9**	**1.63**	**5.51**	**1.25E-05**
**Post-translational protein modification**	5279 79796 9945 29929 5329 79001 29926 5281 4121 811 8818 22872 29880 2677 6675 9695 79868 2530 5238 3998 5277	**99**	**21**	**7.70**	**2.73**	**1.91E-05**
**G2/M Checkpoints**	5984 4175 55388 990 5982 6119 8318 4173 5985 4174 472 1017	**40**	**12**	**3.11**	**3.86**	**3.31E-05**
**Activation of ATR in response to replication stress**	5984 4175 55388 990 5982 6119 8318 4173 5985 4174 1017	**34**	**11**	**2.64**	**4.16**	**3.21E-05**
**DNA strand elongation**	5984 4175 5558 5982 6119 5557 8318 4173 5985 4174	**30**	**10**	**2.33**	**4.29**	**5.50E-05**
**Pyrimidine metabolism**	7371 7084 1890 1633 7372 1806 4907	**18**	**7**	**1.40**	**5.00**	**0.0002**
**Asparagine N-linked glycosylation**	79796 9945 29929 29926 4121 811 8818 22872 29880 6675 9695 79868 2530 5238 3998	**67**	**15**	**5.21**	**2.88**	**0.0002**
**Synthesis of substrates in N-glycan biosythesis**	9945 29926 8818 29880 6675 5238	**14**	**6**	**1.09**	**5.51**	**0.0004**
**G2/M DNA damage checkpoint**	4175 55388 990 8318 4173 4174 472 1017	**27**	**8**	**2.10**	**3.81**	**0.0008**

Supplementary Figure 4. Microarray gene expression analysis.

A

Gene Symbol	Entrez Gene	P-Value	Fold Change
TPM1	7168	1.7E-04	9.85
CDC42	998	9.3E-04	9.52
CELA2A	51032	4.9E-04	7.40
TPM2	7169	3.5E-05	6.93
NPY	4852	7.5E-02	6.61
ITGB1	3688	8.9E-04	6.20
TSPAN8	7103	1.2E-02	5.68
GKN1	56287	1.6E-02	5.46
PDLIM7	9260	1.3E-05	5.30
THBD	7056	2.7E-03	5.03
TNNI3	7137	1.6E-02	4.83
TNNC1	7134	5.5E-03	4.82
KCNB1	3745	3.9E-04	4.64
MYH6	4624	1.5E-01	4.31
PKP4	8502	7.6E-04	4.29
ACTN2	88	2.8E-04	4.17
MICAL2	9645	7.2E-04	4.17
CD36	948	1.5E-02	4.04
GPX3	2878	3.7E-04	3.95
RRAD	6236	2.9E-02	3.94
CRYM	1428	4.5E-03	3.93
TTN	7273	3.3E-02	3.92
PGAM2	5224	1.5E-03	3.72
COL2A1	1280	4.3E-02	3.66
ACE2	59272	1.8E-03	3.62
HIST1H2BG	8339	9.6E-04	3.46
MYH6	4624	1.1E-01	3.46
HCP5	10866	6.8E-03	3.46
MNDA	4332	4.4E-02	3.40
MYH3	4621	6.6E-02	3.33
HIST1H2BC	8339	4.4E-03	3.27
EPB41L1	2036	2.6E-03	3.21
LINC00472	79940	1.3E-03	3.15
HIST2H2BE	8349	3.8E-04	3.15
CDH8	1006	2.2E-04	3.04
PLLP	51090	3.3E-03	3.02
NCAM1	4684	6.1E-04	3.00
GABBR1	2550	1.6E-05	2.95
SYNPO2L	79933	6.0E-02	2.89
SIGLEC15	284266	6.9E-06	2.87
ART4	420	2.7E-03	2.85
MYOZ2	51778	3.5E-03	2.85
CDH1	999	1.2E-03	2.83
OLR1	4973	3.2E-03	2.79
PPL	5493	8.3E-04	2.77
CASQ2	845	2.4E-01	2.69
GNAS	2778	4.0E-03	2.68
JMJD6	23210	1.6E-03	2.63
CELA2B	51032	1.7E-03	2.63
TNNT2	7139	3.2E-02	2.61
RCAN2	10231	5.0E-04	2.60
CLUL1	27098	5.2E-03	2.59
CYP17A1	1586	3.5E-02	2.59
CHI3L1	1116	1.2E-02	2.56
RNF41	10193	6.3E-04	2.55
IL18	3606	5.3E-03	2.53
RAB40B	10966	2.7E-03	2.50
HSD3B1	3283	1.0E-01	2.49
ALDH1A3	220	2.3E-02	2.49
RCAN1	1827	4.3E-02	2.47
OPN3	23596	2.7E-02	2.46
PALMD	54873	7.3E-03	2.46
CD24	1E+08	1.6E-01	2.41
KCNK3	3777	5.4E-02	2.37
SEPT6	23157	2.6E-03	2.32
ST6GALNAC5	81849	1.5E-01	2.31
GPR64	10149	5.6E-02	2.30
NRP1	8829	6.3E-04	2.29
C1orf95	375057	1.0E-02	2.29
BMP4	652	2.9E-03	2.28
CALD1	800	1.8E-03	2.26
HIST1H4H	8365	1.2E-02	2.25
AQP1	358	3.0E-01	2.25
HIST1H2AD	3013	3.5E-03	2.23
GSG1	83445	1.6E-01	2.21
ITGB2	3689	5.9E-02	2.18
DTNA	1837	2.0E-02	2.17
FCGR1B	2210	5.1E-02	2.17
GYPA	2993	1.6E-03	2.15
BAMBI	25805	1.1E-01	2.15
MPZL1	9019	2.5E-03	2.14
ANKRD1	27063	9.9E-03	2.13
OPCML	4978	1.4E-02	2.13
PLAC8	51316	1.2E-02	2.11
DES	1674	5.6E-02	2.10
NECAB2	54550	8.2E-03	2.08
RGS5	8490	6.5E-02	2.07
HTR2B	3357	6.5E-02	2.07
CLDN18	51208	1.6E-03	2.07
CLSTN2	64084	4.6E-02	2.06
HIST2H2AA3	8337	1.6E-03	2.06
PDLIM5	10611	7.8E-02	2.03
EGLN3	112399	1.5E-03	2.03
ARL4C	10123	7.1E-04	2.02
LDB3	11155	7.7E-05	2.01
SLC24A3	57419	2.0E-03	2.01
MEPE	56955	4.2E-03	2.00

B

Gene Symbol	Entrez Gene	P-Value	Fold Change
MFSD9	84804	3.5E-03	-2.00
TNMD	64102	2.3E-02	-2.00
B3GALNT1	8706	9.4E-02	-2.00
HAS2	3037	1.7E-01	-2.03
RAB8B	51762	3.5E-02	-2.04
LY96	23643	4.6E-03	-2.04
PTX3	5806	1.1E-01	-2.04
FBN2	2201	2.4E-03	-2.04
HAPLN1	1404	8.0E-02	-2.06
NTRK2	4915	2.0E-01	-2.07
MAN1A1	4121	2.1E-02	-2.07
PIP5K1B	8395	1.4E-01	-2.08
RABGAP1L	9910	1.1E-02	-2.09
SOCS2	8835	6.5E-02	-2.09
MFAP3L	9848	1.1E-02	-2.10
CPNE3	8895	7.0E-03	-2.11
LRRFIP1	9208	3.8E-03	-2.13
DZIP1	22873	1.2E-03	-2.14
TMEFF1	8577	2.0E-02	-2.14
PNP	4860	2.3E-03	-2.14
MMP3	4314	2.5E-01	-2.15
NEFM	4741	2.3E-01	-2.16
FGF7	2252	5.1E-03	-2.16
RNASE1	6035	1.7E-01	-2.16
LY75	4065	3.9E-03	-2.17
ATF3	467	4.8E-02	-2.18
TM4SF1	4071	8.6E-03	-2.20
CREBL2	1389	1.9E-03	-2.20
PTPRN2	5799	3.2E-03	-2.21
GK	2710	2.7E-02	-2.22
SGPP1	81537	4.4E-03	-2.24
SSX4	6759	1.3E-01	-2.26
VWA5A	4013	4.3E-03	-2.26
DPYD	1806	2.1E-04	-2.27
PLXNC1	10154	2.1E-02	-2.28
SSX1	6756	1.0E-01	-2.29
STOM	2040	3.1E-03	-2.29
KRT19	3880	2.7E-01	-2.29
DPP4	1803	1.1E-02	-2.30
CETN3	1070	4.6E-04	-2.33
CPD	1362	7.3E-05	-2.36
ARL6IP1	23204	1.5E-04	-2.37
GPR183	1880	7.7E-02	-2.38
ID4	3400	1.7E-01	-2.40
SV2A	9900	3.9E-04	-2.40
OGN	4969	2.6E-01	-2.43
FAM184A	79632	3.7E-03	-2.44
DIRAS2	54769	3.9E-01	-2.46
B3GNT2	10678	6.4E-03	-2.52
HLA-DPA1	3113	4.8E-03	-2.57
CTNND1	1500	2.2E-04	-2.59
CSPG5	10675	2.3E-02	-2.61
ATP8B4	79895	2.7E-04	-2.62
CTSK	1513	6.2E-02	-2.69
SPAG1	6674	1.2E-03	-2.69
ERP44	23071	6.2E-03	-2.82
TM4SF4	7104	2.7E-02	-3.64
SCRG1	11341	4.5E-02	-3.93
GCH1	2643	4.4E-03	-3.99

C

Pathway Analysis: GTMMN microRNA Versus GTMMN Upregulated Genes						
**Pathway Name**	**Entrez Gene**	**C**	**O**	**E**	**R**	**P**
Striated Muscle Contraction	7134 7137 7169 1674 4621 7139 88 4624 7168	30	9	0.2	42.14	4.0E-13
Muscle contraction	7134 7137 7169 1674 800 4621 7139 4624 88 7168	45	10	0.3	31.21	5.2E-13
Cell-Cell communication	999 3688 88 4684 51208 1 006	105	6	0.8	8.03	1.0E-04
Smooth Muscle Contraction	7169 800 7168	19	3	0.1	22.18	3.0E-04
Cell junction organization	999 3688 51208 1006	60	4	0.4	9.36	9.0E-04
Integrin cell surface interactions	3689 999 1280 3688	66	4	0.5	8.51	1.2E-03
Cell surface interactions at the vascular wall	3689 4973 3688 7056	70	4	0.5	8.03	1.5E-03
**Pathway Analysis: GTMMN microRNA Versus GTMMN Downregulated Genes**						
**Pathway Name**	**Entrez Gene**	**C**	**O**	**E**	**R**	**P**
Metabolism of nucleotides	1806 4860	50	2	0.2	9.03	2.1E-02

Supplementary Figure 5. Microarray gene expression analysis.

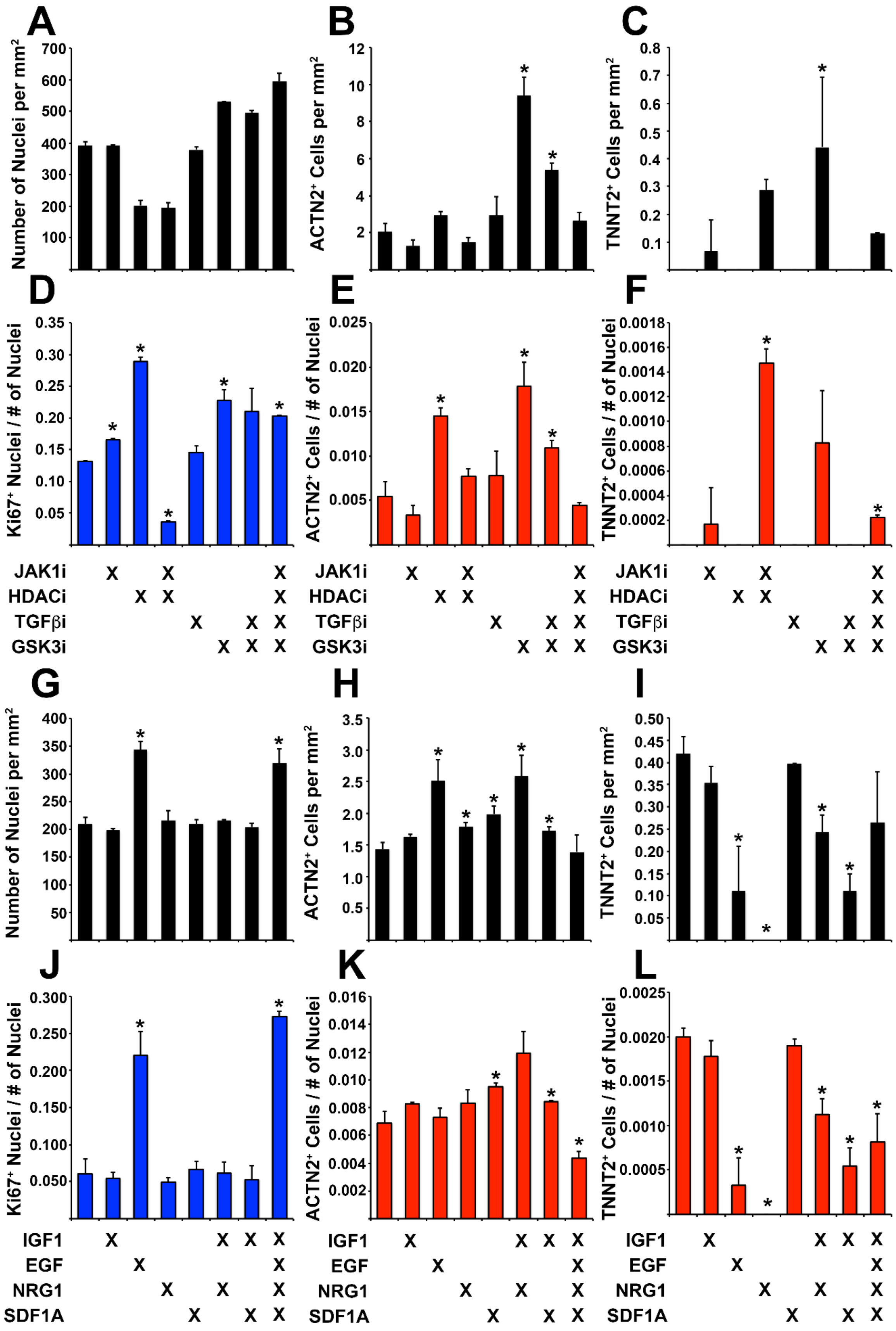

A
B
C
D
E
F
G
H
I
J
K
L
Number of Nuclei per mm^2
ACTN2$^+$ Cells per mm^2
TNNT2$^+$ Cells per mm^2
Ki67$^+$ Nuclei / # of Nuclei
ACTN2$^+$ Cells / # of Nuclei
TNNT2$^+$ Cells / # of Nuclei
JAK1i
HDACi
TGFβi
GSK3i
IGF1
EGF
NRG1
SDF1A

Supplementary Figure 6. Effect of small molecule inhibitors or protein ligands on the transdifferentiation efficiency of HDF transduced with 5 cardiac TF only.

Gene Symbol	Accession Number	Sequence	Amplimer Size
**TNNT2**	NM_000364.2	GGCAGCTGCTGTTCTGAGGGAG	191 bps
		TGCCCTGGTCTCCTCGGTCT	
**MYH6**	NM_002471.2	AGAGGCCCAACGCTCCCTCA	193 bps
		GGCGTTCTTCGCCTTGCCCT	
**MYH7**	NM_000257.2	CCGAGGAGGCGGAGGAGCAA	217 bps
		GCATGGGGCTTTGCTGGCAC	
**MYL2**	NM_000432.3	TTGCTGCCCTTGGGCGAGTG	273 bps
		GGGGGAAGGCGGCGAACATC	
**MYL7**	NM_021223.2	AGCCACCAAGCAGGCCCAAC	172 bps
		TGGGACACTCACCTTCCCCAGC	
**CASQ2**	NM_001232	TCGCCTGCGCCCAGAAGAAAT	205 bps
		CCAGTAGGCAACGAGCAGAGGAA	
**NPPA**	NM_006172.3	GGGCCCCTGGGACTCCTCTG	115 bps
		CCTGTCCATCCTGCCCCCGA	
**ATP2A2**	NM_001681.3	ACCTGGAACCTGCAATACTGGAGTAAC	128 bps
		CCCCAACATCAGTCATGCACAGGG	
**SLC8A1**	NM_001112800	AGTGCCAGACACATTTGCCAGC	129 bps
		GCGATGGACCAGGCCACACC	
**RYR2**	NM_001035.2	AGAGCCGAAGTGTGCAGCGG	213 bps
		CCGCTGGGCCTTGAAGCCAT	
**PLN**	NM_002667	AACTCCCCAGCTAAACACCCGT	167 bps
		ACCAGCAGGACAGGAAGTCTGAA	
**MYH11**	NM_002474	AGTATCACGGGAGAGCTGGA	217 bps
		ATGTCCTCTCGTCTCTGGCT	
**ACTA2**	NM_001613	GCCAAGCACTGTCAGGAATC	213 bps
		GTCACCCACGTAGCTGTCTT	
**COL1A1**	NM_000088	AAGGCCAAGTCGAGGGCCAAGA	246 bps
		TTTCTTGGTCGGTGGGTGACTCTGA	

**COL1A2**	NM_000089	AGGGCCAGGGAAACTTTTGCCG	300 bps
		TCCGGCTGGGCCCTTTCTTACA	
**GATA4**	NM_002052	TCATCTCACTACGGGCACAG	233 bps
		GGGAAGAGGGAAGATTACGC	
**TBX5**	NM_181486	AAAGAATATCCCGTGGTCCC	291 bps
		TTCACTGGGTGATGTCTCCA	
**MEF2C**	NM_002397	ACACCGCCAGCGCTCTTCAC	237 bps
		CCTCGTGGCGCGTGTGTTGT	
**MYOCD**	NM_153604	CAAGCCAAAGGTGAAGAAGC	177 bps
		TAGCTGAATCGGTGTTGCTG	
**NKX2-5**	NM_004387	CCTCAACAGCTCCCTGACTC	201 bps
		GGGGACAGCTAAGACACCAG	
**ACTB**	NM_001101	ACAGAGCCTCGCCTTTGCCGAT	196 bps
		CATGCCCACCATCACGCCCTG	

Supplementary Table 2. Primers used for gene expression analysis.
